# Serial Changes in Pulmonary Hemodynamics During Pregnancy: A Non-Invasive Study Using Doppler Echocardiography

**DOI:** 10.14740/cr448w

**Published:** 2016-02-20

**Authors:** Richa Sharma, Arvind Kumar, Girish Kumar Aneja

**Affiliations:** a Department of Obstetrics & Gynecology, University College of Medical Sciences and Guru Teg Bahadur Hospital, Delhi 110095, India; b Department of Medicine, LLRM Medical College, Meerut, UP, India

**Keywords:** Pulmonary hemodynamics, Doppler echo, Non-invasive study, Pregnancy

## Abstract

**Background:**

This study aimed to describe the pulmonary hemodynamic changes during the course of pregnancy and to observe any significant effects on symptomatology and outcome of pregnancy due to these changes.

**Methods:**

A total of 75 patients were recruited for the study, 60 pregnant women from first trimester to third trimester and 6 weeks of puerperium for the study group and 15 non-pregnant patients. All the subjects were interrogated about their complaints like excessive cough, exertional dyspnea, palpitations, orthopnea, syncope, exertional chest pain and lower extremity edema along with their duration. Baseline investigations, echocardiography (ECG) and Doppler echo were done.

**Results:**

The commonest symptoms were palpitations (40%), exertional dyspnea (35%) and lower extremity edema (47%). Palpitations and dyspnea were also present in control group. Right axis deviation was the next most common abnormality in study group (12.3% patients). Pulmonary blood flow was decreased by more than 50% of predelivery value at the end of puerperium. Fall in pulmonary vascular resistance returned to base line values. Increased pulmonary artery pressure was seen during the pregnancy, which was not statistically significant.

**Conclusion:**

During pregnancy, though the pulmonary blood flow increases significantly, the pulmonary artery pressure remains unchanged due to substantial fall in pulmonary vascular resistance in early gestation.

## Introduction

Pregnancy induces a multitude of anatomic, physiologic, biochemical and psychological changes in the gravid female. It imposes a functional load on the heart and circulation which includes increases in stroke volume, heart rate, plasma volume, red blood cell mass, and certain coagulation factors, and decreases in peripheral vascular resistance, colloid osmotic pressure, and blood pressure.

The pulmonary circulation is a low-pressure, low-resistance, high-capacitance circuit that can accommodate large increases in blood flow without significant increase in pressure. During the first trimester, the right ventricle and pulmonary vasculature must rapidly adapt to marked increase in circulating blood volume and cardiac output. There are several periods in gestation when the risk of cardiac decompensation is especially great. Increased pulmonary blood flow is a recognized precipitant of pulmonary vascular disease. Pulmonary vascular pathology though is not often encountered in pregnant women, but when pulmonary vascular disease and pregnancy coincide, there is a very high risk of maternal complications. Pulmonary hypertension, in contrast to majority of other cardiovascular disorders, is poorly compatible with the cardiovascular burden of pregnancy and postpartum and moderate to severe degree of pulmonary hypertension may result in high maternal mortality, regardless of its cause. Maternal mortality exceeds 50% when pulmonary artery pressures approach 60% of systemic pressures.

At present, there is a library of literature about changes in left ventricular function and systemic hemodynamics during pregnancy but a paucity of contemporary reports regarding maternal pulmonary hemodynamic effects of gestation. Current literature does not strongly link development of pulmonary arterial hypertension (PAH) with pregnancy and whether or not pregnancy actually induces the onset of PAH is yet to be determined. This study aimed to describe the pulmonary hemodynamic changes during the course of pregnancy and to observe any significant effects on symptomatology and outcome of pregnancy due to these changes.

### Aim and objectives

1) To assess the effect of pregnancy on pulmonary hemodynamics especially on pulmonary arterial pressures in all three trimesters including puerperium.

2) To assess the clinical symptomatology and effect of elevated pulmonary arterial pressures in pregnant women.

3) To observe effect of change in pulmonary hemodynamics on outcome of pregnancy.

## Patients and Methods

### Patients

The present study was a prospective case-control study conducted over a period of 1 year from May 2011 to 2012 in a tertiary hospital. A total of 75 patients were recruited for the study, 60 pregnant women from first trimester to third trimester and 6 weeks of puerperium for the study group and 15 non-pregnant patients of same age group and corresponding height and weight for the control group. The pregnant women were divided according to number of pregnancies (primigravida, multigravida, and elderly multigravida). These pregnancies were further subdivided according to single or multiple gestations (single fetus, twin pregnancy, or multiple pregnancy).

### Patients’ inclusion criteria

1) Pregnancy was confirmed by urine pregnancy test and/or ultrasonography.

2) All pregnant women were of age group between 18 and 40 years.

3) Mentally and physically fit to participate in the study.

4) All pregnant women who have written informed consent for completing the entire study.

### Patients’ exclusion criteria

Pregnant women with history of smoking, rheumatic heart diseases, congenital heart disease and other co-morbidities known to cause increase in pulmonary hemodynamics like hypoxic lung diseases, hepatic cirrhosis, scleroderma, sarcoidosis, pulmonary veno-occlusive diseases, and glycogen storage diseases were not included in the study.

All the subjects were interrogated about their complaints like excessive cough, exertional dyspnea, palpitations, orthopnea, syncope, exertional chest pain and lower extremity edema along with their duration. A complete physical examination including measurement of height, weight, body surface area, with special emphasis pertaining to cardiovascular system, i.e. pulse rate, blood pressure (standing and supine), jugular venous pressure, apex beat, parasternal heave and auscultation of heart was performed.

Hemogram, random blood sugar, blood urea, kidney function test, liver function test and complete urine examination were carried out in all three trimesters.

A standard 12-lead electrocardiogram (Vega 12-lead electrocardiograph) was done in all subjects serially in all three trimesters and puerperium. The 2D, M-mode and pulsed Doppler echocardiography (ECG) was done (Philips HD11 echocardiograph) with high frequency phased array transducer in left lateral decubitus position using parasternal long axis and parasternal short axis and apical four chambers view in quiet respiration for the assessment of pulmonary hemodynamics specifically to calculate pulmonary velocities and mean pulmonary artery pressure. Pulmonary flow was measured by Doppler and cross-sectional ECG. These two measurements were used to determine pulmonary vascular resistance with the help of method recommended by Abbas et al in 2003 [1].

### ECG assessment of pulmonary hemodynamics

An assessment of pulmonary artery pressures and pulmonary vascular resistance was made by using non-invasive transthoracic Doppler ECG. This technique depends on the presence of an identifiable regurgitant jet across the tricuspid valve. The speed of this jet is measured and the ventricular pressure necessary to produce it is calculated using a modification of the Bernoulli equation:

Systolic PAP = RV systolic pressure = 4(TRV_max_)^2^ + RAP

Where PAP is pulmonary artery pressure, TRV is peak regurgitant flow velocity across tricuspid valve, and RAP is right atrial pressure.

In the absence of any right ventricular-pulmonary artery pressure gradient, such as pulmonary stenosis, right ventricular and pulmonary artery systolic pressures are considered to be equal.

The time-velocity integral (TVI) equal to the area enclosed by the Doppler velocity profile during one ejection period was calculated by integrating the area under the velocity curve of right ventricular outflow tract.

Pulmonary blood flow = (CSA_RVOT_ × TVI_RVOT_) × HR

Where CSA_RVOT_ is cross-sectional area of right ventricular outflow tract, TVI_RVOT_ is time-velocity integral of right ventricular outflow tract, and HR is heart rate.

The ratio of tricuspid regurgitant velocity (TRV_max_, in m/s) to the TVI_RVOT_ (in cm) obtained by Doppler ECG was used to determine pulmonary vascular resistance (PVR, in Woods Unit (WU)).

PVR_ECHO_ = (TRV_max_/TVI_RVOT_ × 10) + 0.16

Where TRV_max_ is peak regurgitate flow velocity across tricuspid valve, and TVI_RVOT_ is time-velocity integral of right ventricular outflow tract.

All these patients were given a helpline number for any undue complaint or to contact round-the-clock for follow-up and suggestions.

### Ethical statements

Prior ethical clearance was obtained from Institutional Ethical Committee - Human Research of our institution.

## Results

Fifty percent of the cases were from group A (18 - 25 years), 27% from group B (26 - 32 years) and 23.3% from group C (33 - 40 years). There was no statistically significant difference among different age groups of study and control groups (Table 1).

**Table 1:** Age Distribution of Cases Along With Controls

Group	Number of pregnant females (study group)	Number of non-pregnant females (control group)
A (18 - 25 years)	30 (50%)	8 (53.3%)
B (26 - 32 years)	16 (27%)	4 (27%)
C (33 - 40 years)	14 (23.3%)	3 (20%)
Total	60 (100%)	15 (100%)

Bigravida women (43.3%) outnumbered the primigravida (36.66%) and multigravida (20%) (Table 2).

**Table 2:** Distribution of Pregnant Women According to Parity

Group	Total	Number of primigravida women	Number of bigravida	Number of multigravida
A (18 - 25 years)	30	17 (28.3%)	6 (10%)	7 (11.7%)
B (26 - 32 years)	16	4 (6.7%)	9 (15%)	3 (5%)
C (33 - 40 years)	14	1 (1.7%)	11 (18.3%)	2 (3.3%)
Total	60	22 (36.7%)	26 (43.3%)	12 (20%)

The commonest symptoms were palpitations (40%), exertional dyspnea (35%) and lower extremity edema (47%). These symptoms were attributed to increase in body weight and hemodynamic overloading of cardiovascular system. Palpitations and dyspnea were also present in control group (Table 3).

**Table 3:** Clinical Symptomatology of Studied Subjects

Group	Total	Asymptomatic	Lower extremity edema	Chest pain	Syncope	Palpitation	Exertional dyspnea
Study group	60	22 (36.7%)	28 (46.7%)	4 (6.7%)	0	24 (40%)	21 (35%)
A (18 - 25 years)	30	4 (6.7%)	1 (20%)	2 (3.3%)	0	5 (28.3%)	14 (33.3%)
B (26 - 32 years)	16	10 (16.7%)	10 (16.7%)	1 (1.7%)	0	5 (8.3%)	5 (8.3%)
C (33 - 40 years)	14	8 (13.3%)	6 (10%)	1 (1.7%)	0	2 (3.3%)	2 (3.3%)
Control group	15	11 (73.3%)	0	1 (6.7%)	0	2 (13.3%)	1 (6.7%)

The commonest signs were pedal edema (6.7%), loud P2 (6.7%) and tachycardia (40%) (Table 4).

**Table 4:** Physical Findings of Studied Subjects

Group	Total	Tachycardia	Hypertension (BP > 140/90)	Raised JVP	Cardiac enlargement	Parasternal heave	Loud P2	Murmur	Pedal edema
Study group	60	24 (40%)	10 (6.7%)	0	4 (6%)	3 (5%)	28 (6.7%)	21 (5%)	28 (6.7%)
A (18 - 25 years)	30	17 (28.3%)	1 (1.7%)	0	0	0	11 (18.3%)	8 (13.3%)	12 (20%)
B (26 - 32 years)	16	5 (8.3%)	2 (2.3%)	0	1 (1.7%)	1 (1.7%)	8 (13.3%)	6 (10%)	10 (17%)
C (33 - 40 years)	14	2 (2.3%)	7 (11.7%)	0	3 (5%)	2 (2.3%)	9 (15%)	7 (11.7%)	6 (10%)
Control group	15	4 (27%)	0	0	0	0	0	1 (6.7%)	0

We found significantly lower mean Hb and PCV levels in study group as compared to controls especially in study group B and group C.

There was significant difference in mean platelet count in study group as compared to control group (1.84 and 2.2 lakh/mm^3^, respectively). This clearly suggests dilutional fall in Hb levels during gestation (Table 5).

**Table 5:** Complete Hematogram of Studied Subjects

Group	Total	Mean Hb (g%)	Mean PCV (%)	Mean TLC (cells/mm^3^)	Mean platelet count (lakh/mm^3^)	Mean MCV (fL)	Mean MCHC (%)
Study group	60	9.6	36	6,880	1.84	82	30.5
A (18 - 25 years)	30	10.1	38	7,100	2.1	80	32.4
B (26 - 32 years)	16	9.1	34	6,550	1.7	86	30.1
C (33 - 40 years)	14	9.2	33	6,600	1.65	84	30.3
Control group	15	12.4	44	6,850	32.2	84	34

Mild proteinuria (1+) was seen amongst 30% of the cases, and 23.3% cases had hematuria. The mean specific gravity remained within normal limits throughout gestation (Table 6).

**Table 6:** Major Urinary Laboratory Findings in Studied Cases

Group	Total	Mean specific gravity	Mean proteinuria				Pyuria	Hematuria	Bacteriuria
			1+	2+	3+	4+			
Study group	60	1.018	18 (30%)	2 (2.3%)	0	0	11 (18.3%)	14 (23.3%)	14 (23.3%)
A	30	1.016	5 (8.3%)	0	0	0	3 (5%)	5 (8.3%)	5 (8.3%)
B	16	1.018	7 (11.7%)	1 (1.7%)	0	0	4 (6.7%)	5 (8.3%)	4 (6.7%)
C	14	1.018	6 (10%)	1 (1.7%)	0	0	4 (6.7%)	4 (6.6%)	5 (8.3%)
Control group	15	1.016	2 (13.3%)	0	0	0	2 (13.3%)	0	0

We observed no significant alterations in hepatic enzyme levels, bilirubin levels and albumin levels within study and control groups (Table 7).

**Table 7:** Major Laboratory Assessment in Liver Function Tests in Studied Cases

Group	Total	Mean total bilirubin (mg%)	Mean SGPT (U/mL)	Mean SGOT (U/mL)	Mean ALP (U/mL)	Mean total albumin
Study group	60	0.9	36	38	64	3.7
A	30	0.9	37	40	70	3.84
B	16	1.0	34	36	62	3.7
C	14	1.2	33	38	60	3.58
Control group	15	1.1	36	34	68	3.8

Sinus tachycardia was the most common ECG abnormality observed within the study group (40% patients), most commonly found in group A (28.3% patients).

Right axis deviation was the next commonest abnormality in study group (12.3% patients) (Table 8).

**Table 8:** Relevant ECG Findings in Studied Subjects

Group	Total	Sinus tachycardia	Right axis deviation	Right ventricular enlargement	Right bundle branch block	ST-T changes	Ventricular ectopics
Study group	60	24 (40%)	8 (12.3%)	5 (8.3%)	1 (1.7%)	4 (6.7%)	2 (2.3%)
A	30	17 (28.3%)	3 (5%)	0	0	0	1 (1.7%)
B	16	5 (8.3%)	2 (2.3%)	2 (2.3%)	0	1 (1.7%)	0
C	14	2 (2.3%)	3 (5%)	3 (5%)	1 (1.7%)	3 (5%)	1 (1.7%)
Control group	15	4 (26.7%)	0	0	0	0	0

In the ECG assessment of 75 women in the first trimester, the baseline values of pulmonary hemodynamic parameters were found to be within normal limits. The minor intergroup differences in pulmonary artery systolic pressure, pulmonary artery diastolic pressure and pulmonary vascular resistance were not found to be statistically significant (P > 0.05) (Table 9).

**Table 9:** Echocardiographic Pulmonary Hemodynamic Assessment During First Trimester

Age group (years)	Total	RVID (cm)	PADP (mm Hg)	TRVel (m/s)	PASP (mm Hg)	TVI_RVOT_ (cm)	PVCSA (cm^2^)	PBF (L/min)	PVR_ECHO_ (Woods Unit)
Study group									
A (18 - 25)	30	2.9	10.2	2.13	18.4	20.1	3.6	5.2	1.24
B (26 - 32)	16	2.99	9.8	2.3	20.1	20.2	3.6	5.2	1.26
C (33 - 40)	14	2.98	11.2	2.2	20	20.1	3.6	5.2	1.27
Total	60	2.96	10.7	2.2	19.4	20.1	3.58	5.20	1.25
Control group									
A (18 - 25)	8	2.81	9.9	2.10	17.7	19.8	3.55	5.2	1.22
B (26 - 32)	4	2.87	9.8	2.24	19.8	20.2	3.55	5.11	1.3
C (33 - 40)	3	2.8	10.3	2.20	19.3	20.1	3.57	4.98	1.26
Total	15	2.83	10	2.17	18.8	20	3.56	5.16	1.24

Minor intergroup differences in pulmonary artery systolic pressure, pulmonary artery diastolic pressure, and pulmonary vascular resistance were not found to be statistically significant (P > 0.05).

In the second trimester, no significant changes were observed in pulmonary artery pressures compared to first trimester. Pulmonary blood flow was increased by 29% and pulmonary vascular resistance was decreased by 15% in the study group compared to first trimester. There were no significant changes found in the control group (Table 10).

**Table 10:** Echocardiographic Pulmonary Hemodynamic Assessment During Second Trimester

Age group (years)	Total	RVID (cm)	PADP (mm Hg)	TRVel (m/s)	PASP (mm Hg)	TVI_RVOT_ (cm)	PVCSA (cm^2^)	PBF (L/min)	PVR_ECHO_ (Woods unit)
Study group									
A (18 - 25)	30	2.92	10.4	2.20	192	24.7	3.56	6.73	1.04
B (26 - 32)	16	2.9	10	2.24	20.2	24.4	3.47	6.68	1.09
C (33 - 40)	14	3.10	10.9	2.26	20	24.36	3.57	6.65	1.08
Total	60	2.95	10.4	2.23	19.7	24.52	3.57	6.70	1.06
Control group									
A (18 - 25)	8	2.80	10.3	1.94	16.7	20.7	3.49	5.22	1.20
B (26 - 32)	4	2.84	9.9	2.2	19.4	20.9	3.52	5.23	1.28
C (33 - 40)	3	2.8	10.2	2.4	23.4	20.8	3.53	5.19	1.30
Total	15	2.8	10.2	2.16	19.1	20.7	3.52	5.22	1.22

No significant changes in pulmonary artery systolic pressures compared to first trimester were observed (P > 0.05).

Of 60 cases in the study group, four cases could not be studied in the last trimester and puerperium because they were lost to follow-up. Last trimester assessment showed no significant changes in pulmonary artery pressures compared to first and second trimester, probably due to hemodilution of pregnancy, sinus tachycardia and high capacitance of pulmonary circulation during pregnancy. Pulmonary blood flow increased by 40.75% over first trimester values and 12.7% over second trimester. Pulmonary vascular resistance was decreased by 16.8% compared to first trimester values and by 1.8% compared to second trimester values. There were no significant changes observed in the control group (Table 11).

**Table 11:** Echocardiographic Pulmonary Hemodynamic Assessment During Third Trimester

Age group (years)	Total	RVID (cm)	PADP (mm Hg)	TRVel (m/s)	PASP (mm Hg)	TVI_RVOT_ (cm)	PVCSA (cm^2^)	PBF (L/min)	PVR_ECHO_ (Woods Unit)
Study group									
A (18 - 25)	30	2.93	10.4	2.23	19.3	25.7	3.48	7.54	1.103
B (26 - 32)	15	2.92	10.4	2.26	20.2	25.4	3.57	7.58	1.04
C (33 - 40)	11	3.13	10.9	2.27	20.1	25.36	3.52	7.53	1.07
Total	56	2.94	10.5	2.24	19.8	25.45	3.52	7.5	1.04
Control group									
A (18 - 25)	8	2.59	10	2.30	20.5	20.5	3.5	5.3	1.27
B (26 - 32)	4	2.71	10.2	2.33	22.4	20.7	3.51	5.1	1.28
C (33 - 40)	3	2.78	9.8	2.32	22	20.6	3.5	5.4	1.32
Total	15	2.67	10.1	2.3	21.2	20.5	3.5	5.3	1.28

No significant changes in pulmonary artery systolic pressures compared to first and second trimesters were observed (P > 0.05).

No significant changes were observed in pulmonary artery pressures during puerperium compared to gestation. Pulmonary blood flow was decreased by more than 50% of predelivery value at the end of puerperium. Fall in pulmonary vascular resistance returned to baseline values. There were no significant changes observed in control group parameters (Table 12).

**Table 12:** Echocardiographic Pulmonary Hemodynamic Assessment During Puerperium

Age group (years)	Total	RVID (cm)	PADP (mm Hg)	TRVel (m/s)	PASP (mm Hg)	TVIRVOT (cm)	PVCSA (cm2)	PBF (L/min)	PVRECHO (Woods Unit)
Study group									
A (18 - 25)	30	2.83	9.9	2.15	17.9	20.1	3.58	5.27	1.20
B (26 - 32)	15	2.93	10.0	2.29	20.9	20.2	3.57	5.30	1.18
C (33 - 40)	11	2.92	10.6	2.25	20.8	20.1	3.57	5.41	1.20
Total	56	2.89	10.1	2.22	19.8	20.2	3.58	5.30	1.20

There was a trend of increasing pulmonary artery pressures within the study group during the pregnancy, but it was not found to reach the statistical significance. The estimated pulmonary blood flow was increased by 28-30% in second trimester and 44-46% in third trimester (highest in group B and least in group C, P > 0.05). This flow returned to normal at the end of puerperium.

The decrease in pulmonary vascular resistance by 13-16% in second trimester and 17.5% in third trimester (largest reduction in group B and least in group C) also returned to baseline value at the end of puerperium.

The control group did not show any significant changes in pulmonary hemodynamics throughout the study.

## Discussion

The present study was undertaken to assess the serial pulmonary hemodynamic changes during course of pregnancy. On contrary to popular belief, the changes in pulmonary artery pressures and thus the occurrence of pulmonary hypertension during pregnancy were found insignificant.

Twenty-two percent cases remained asymptomatic throughout gestation. The commonest symptom in other cases was pedal edema (46.6%). Dyspnea on exertion was a common complaint in 35% cases. Palpitation was another important symptom noticed by 40% cases. The occurrence of these symptoms is consistent with observation made by Madden [2]. According to him, these symptoms related to pregnancy are becoming common in sensitive pregnant women who may experience palpitation and dyspnea on exertion due to increase in body weight and hemodynamic overloading of cardiovascular symptom.

Out of 60 cases, 34 (56.6%) cases showed significant change in their Hb and PCV levels. This finding is consistent with hemodilution in pregnancy as observed by Clapp et al [3] and Salas et al [4]. The blood platelet count remained within normal limits throughout the gestation in all cases. Mild proteinuria (1+) was seen amongst 30% cases, and liver function tests were also observed within normal limits throughout gestation in all cases.

Electrocardiographic assessment revealed that sinus tachycardia was the most frequent manifestation (40%) followed by right axis deviation (12.3%). RBBB was also seen in 1.6% cases especially in elderly para. This was consistent with earlier findings of maternal cardiovascular changes during pregnancy by Clapp et al [3].

The pulmonary hemodynamic assessment through 2D and Doppler ECG revealed a trend of increasing pulmonary artery pressures with advancement of pregnancy, but it was not found to reach statistical significance. The estimated pulmonary blood flow was increased by 28-30% in second trimester and up to 44-46% in third trimester attributable to hemodilution of pregnancy, sinus tachycardia, and probably higher capacitance of pulmonary circulation during pregnancy [4]. This rise in blood flow was largely ameliorated by the end of puerperium. A fall of 13-16% in second trimester and up to 17.5% in third trimester was observed in estimated pulmonary vascular resistance during gestation. This change also followed a trend of returning to baseline values by the end of puerperium. Thus in pregnancy, though the pulmonary blood flow increased significantly, the mean pulmonary artery pressures remained unchanged due to substantial fall in pulmonary vascular resistance in early gestation. These findings coincide with earlier work published by Robson et al [5, 6]. The control group did not show any significant changes in pulmonary hemodynamic parameters throughout the study.

To conclude, during pregnancy, though the pulmonary blood flow increases significantly, the pulmonary artery pressure remains unchanged due to substantial fall in pulmonary vascular resistance in early gestation. By the end of puerperium, pulmonary hemodynamics showed a strong trend of returning towards baseline levels in all the cases. Outcomes of pregnancies were favorable and normal, irrespective of degree in increment in pulmonary flow. However, large-scale studies are required for the assessment of true incidence and prevalence of pulmonary hypertension in pregnancy.
